# Prognostic Significance and Molecular Mechanism of ATP-Binding Cassette Subfamily C Member 4 in Resistance to Neoadjuvant Radiotherapy of Locally Advanced Rectal Carcinoma

**DOI:** 10.1371/journal.pone.0085446

**Published:** 2014-01-15

**Authors:** Zhiqi Yu, Chang Zhang, Rui Chai, Yan Du, Xianhua Gao, Junjie Xing, Enda Yu, Wei Zhang, Xiaoqing Zhang, Guangwen Cao, Chuangang Fu

**Affiliations:** 1 Department of Colorectal Surgery, Changhai Hospital, Second Military Medical University, Shanghai, China; 2 Department of Epidemiology, College of Basic Medical Sciences, Second Military Medical University, Shanghai, China; 3 Department of Radiotherapy, Changhai Hospital, Second Military Medical University, Shanghai, China; 4 Department of Colorectal Surgery, Zhejiang Provincial People's Hospital, Hangzhou, Zhejiang, China; University of California Davis, United States of America

## Abstract

**Background:**

Mechanism of radioresistance in rectal carcinoma remains largely unknown. We aimed to evaluate the predictive role of ATP-binding cassette subfamily C member 4 (ABCC4) in locally advanced rectal carcinoma and explore possible molecular mechanisms by which ABCC4 confers the resistance to neoadjuvant radiotherapy.

**Methods:**

The expression of ABCC4 and P53 mutant in biopsy tissue specimens from 121 locally advanced rectal carcinoma patients was examined using immunohistochemistry. The factors contributing to 3-year overall survival and disease-free survival were evaluated using the Kaplan-Meier method and Cox proportional hazard model. Lentivirus-mediated small hairpin RNA was applied to inhibit ABCC4 expression in colorectal carcinoma cell line RKO, and investigate the radiosensitivity in xenograft model. Intracellular cyclic adenosine monophosphate concentration and cell cycle distribution following irradiation were detected.

**Results:**

High expression of ABCC4 and p53 mutant in pretreated tumors, poor pathological response, and high final tumor staging were significant factors independently predicted an unfavorable prognosis of locally advanced rectal carcinoma patients after neoadjuvant radiotherapy. Down-regulation of ABCC4 expression significantly enhanced irradiation-induced suppression of tumor growth in xenograft model. Furthermore, down-regulation of ABCC4 expression enhanced intracellular cyclic adenosine monophosphate production and noticeable deficiency of G1-S phase checkpoint in cell cycle following irradiation.

**Conclusions:**

Our study suggests that ABCC4 serves as a novel predictive biomarker that is responsible for the radioresistance and predicts a poor prognosis for locally advanced rectal carcinoma after neoadjuvant radiotherapy.

## Introduction

Treatment of locally advanced rectal carcinoma (LARC, T3-4 or/and N1-2 lesions) remains to be a challenge [1]. No great improvement have been made over years. Although the current multidisciplinary treatment strategy recommends preoperative neoadjuvant radiotherapy (nRT) because of its significant benefits in decreasing tumor stage, increasing the opportunities of curative resection and sphincter preservation, improving the local control, and even achieving clinical or pathological complete regression in the short term [2], there is no enough evidence to support that nRT significantly prolong the long-term survival rate in LARC patients, which largely restricts its clinical use [3]. Understanding the molecular basis of individual heterogeneity in the susceptibility to radiotherapy and identifying predictive and prognostic markers from pretreatment biopsy specimens are essential to select LARC patients who are more likely to benefit from nRT, thus improving their long-term prognosis.

Our previous studies have demonstrated a combined predictive value of p53 and p21 for tumor regression after preoperative nRT in rectal carcinoma patients and identified that ATP-binding cassette subfamily C member 4 (ABCC4) may play a role in response to irradiation of CRC cell line HT29 *in vitro* [4]–[5]. ABCC4 has been identified as an active ATP-dependent export pump belonging to the ATP-binding cassette transporter superfamily for exporting cyclic nucleotides and organic anions across extra- and intra-cellular membranes [6]. ABCC4 confer topoisomerase II inhibitor-resistance in macrophages and also serve as a critical member of the multidrug resistance-associated protein subfamily involved in efflux of anticancer/antiviral nucleotide agents inducing resistance to chemotherapy in several kinds of carcinomas [7]–[8]. ABCC4 is distinct from the other subfamily members by its dual membrane localization in polarized cell types [9]. Although the functional role of ABCC4 has not been well clarified in colorectal diseases, expression of ABCC4 has been detected in several CRC cell lines [10].

According to our previous data, the positive rate of ABCC4 expression in pretreatment tissue specimens of LARC was substantial while ABCC4 expression in biopsy specimens was significantly associated with short-term pathological response to nRT in LARC patients [5]. In the present study, we aimed to evaluate the long-term prognostic significance of ABCC4 in this group of LARC patients treated with nRT. We also assess the role of ABCC4 on radioresistance in the xenograft model. This study provides evidence indicating that ABCC4 is a useful predictive marker for LARC prognosis, and a novel therapeutic target for enhancing radiosensitivity and ultimately developing more effective individualized therapies.

## Materials and Methods

### Ethics Statement

The study protocol conformed to the 1975 Declaration of Helsinki Principles and was approved by the human ethic committee of Changhai Hospital and Second Military Medical University (Shanghai, China). All animal treatments were strictly in accordance with international ethical guidelines concerning the Care and Use of Laboratory Animals, and the experiments were carried out with the approval of the Committee of Experimental Animal Administration of the Second Military Medical University. Written informed consent was provided from all patients participating in this study.

### Patients

Biopsy tissue specimens were obtained from 121 LARC patients with a mean age of 57.8 (SD = 11.2) years, as previously described [5]. Pretreatment biopsy was examined to confirm the diagnosis of rectal adenocarcinoma. Pelvic CT/MRI scans were used to stage the clinical tumors as T3/T4 N 0 to 2 and M0. The tumors were located within 12 cm from the anal verge as confirmed by digital rectal examination and flexible sigmoidoscopy. After the first biopsy, the patients underwent long-course preoperative nRT (a total dose of 45–50.4 Gy of irradiation to the pelvic region in 25–28 fractions over a period of 5 weeks). Six to 8 weeks after nRT, the patients received radical surgery (low anterior resection, LAR or abdominoperineal resection, APR) following the principle of total mesorectal excision in the Department of Colorectal Surgery, Changhai Hospital (Shanghai, China). We collected postoperative pathology reports and further determined Circumferential Resection Margin (CRM) status and final tumor staging according to ypTNM staging criterion. The patients were then treated with the standard FOLFOX4 therapeutic regimen (bolus plus continuous-infusion fluorouracil plus leucovorin and oxaliplatin) for 6 to 8 cycles after surgery. All patients were routinely followed up every 3 months on an outpatient bases and/or by telephone calls according to our standard registration and follow-up system [11]. The last follow-up date was December 31, 2012. Prognosis was estimated as 3-year overall survival (OS) and 3-year disease-free survival (DFS).

### Immunohistochemistry

The expression of ABCC4 and p53 protein in each pretreatment biopsy tissue specimen was detected by immunohistochemistry according to our previous protocol [12]. Antibodies were diluted to 1∶1000 for ABCC4 protein and 1∶200 for p53 protein (Cell Signaling Technology, Beverly, MA). Scoring the intensity of immunostaining was done semiquantitatively, and immunostaining was scored based on the percentage of the stained tumor cells: 0–10% as negative (−), 11–25% as slightly positive (+), 26–50% as moderately positive (++), and 51-100% as strongly positive (+++), respectively. All specimens were analyzed independently by two senior pathologists who were blinded to the clinical information. There was close agreement (>90%) among the two pathologists. Disagreements were resolved by consensus.

### Cell Lines and RNA Interference

Human CRC cell line RKO was purchased from the Institute of Cell Biology, Chinese Academy of Science (Shanghai, China). Three different silencing sequences for short hairpin RNA (shRNA) were designed according to previous report [5]. After screening, the most effective sequence of ABCC4 shRNA 5′-GCACAGAAGCCTTCTTTAA-3′ was chosen, inserted into the lentiviral vector, and then transfected into RKO cells as previously described [13]. Nonsilencing sequence 5′-TTCTCCGAACGTGTCACGT-3′ was used as a control. Cells were separated into positive experiment group (RKO-KD), negative control group (RKO-NC), and blank control group (RKO-CON). All cells were routinely cultured, and the knockdown efficiency was confirmed at both RNA and protein levels by real-time RT-PCR and western blot assay, as described in our previous protocol [5].

### Xenograft Model

Five-six weeks old male athymic nude BALB/c mice were maintained under pathogen-free conditions, and then stratified into 3 groups (10 mice/group). Thereafter, 1×10^6^ of RKO-KD, RKO-NC and RKO-CON cells suspended in 100 µl PBS were subcutaneously inoculated into the left armpit of mouse, respectively. We then chose mouse with tumor volume of 0.20 cm^3^ on average and grouped (6 mice/group) for subsequent experiment. Tumor volume was calculated according to the following formula: V = 0.4×D×d^2^ (V, volume; D, longitudinal diameter; d, latitudinal diameter). The mice in experiment groups [RKO-KD (+), RKO-NC (+), RKO-CON (+)] received irradiation with a total dose of 10 Gy in 5 fractions over 5 consecutive days to the tumor area with the rest of the body shielded, while those in control groups [RKO-KD (−), RKO-NC (−), RKO-CON (−)] did not receive irradiation. Tumor growth was monitored and measured every 3 days for 3 weeks, then the mice were sacrificed and the tumors were weighed.

### Irradiation

In order to simulate the radiotherapy condition in clinical setting, medical 6-megavolt x-ray linear accelerator (Clinac 21EX, Varian Medical Systems, Palo Alto, CA) was utilized to perform irradiation to both cell samples and xenograft model at room temperature. The irradiation conditions were: treatment field = 30 cm×30 cm, ounce-skin distance = 100 cm, and radiation dose rate = 400 cGy/min.

### Detection of Intracellular Cyclic Adenosine Monophosphate (cAMP)

Prior to performing cAMP detection assay, RKO-KD, RKO-NC, and RKO-CON cells were exposed to irradiation with a single dose of 4 Gy, and then cultured for 3, 6, 9, and 12 hrs, respectively. Cells without irradiation were used as controls. According to the manufacturer's instructions, 20 µl cAMP-Glo lysis buffer (Promega, Madison, WI) was added to each cell well to release cAMP. The cell plates were then incubated with constant shaking at room temperature for 15 min. cAMP detection solution (40 µl), which contained protein kinase A (PKA), was then added and mixed by shaking for 60 sec. After the cell plates were incubated at room temperature for another 20 min, 80 µl Kinase-Glo reagent was added to terminate the PKA reaction at room temperature for 10 minutes. Relative luminescence units (RLU) were measured in each well using a plate-reading luminometer (Bio-Rad, Nashua, NH).

### Detection of Cell Cycle Distribution

Cell cycle of those with or without irradiation was examined using cytometry. Exponentially growing cells were digested, collected, washed twice with ice-cold PBS, fixed in cold 75% ethanol overnight at 4°C, then washed and re-suspended in 500 µl PBS containing a concentration of 10 mg/ml RNase A (Sigma, St Louis, MO), and finally stained with 150 µl Propidium Iodide kit (Sigma, St Louis, MO) for 30 min at room temperature in the dark. DNA content was detected by a FACScan flow cytometer (Miltenyi, Germany) to analyze the relative proportion of cells in G0/G1, S and G2/M phases.

### Statistical Analysis

Differences in categorical variables were examined using χ^2^ test. Pearson's r test was applied to evaluate the correlations of mRNA level with protein level of ABCC4 in fresh tissue samples. The OS and DFS were calculated using the Kaplan-Meier method. We recruited all important clinicopathological features into the univariate log-rank survival analysis model, further recruited factors whose *P* value less than 0.10 into multivariate Cox regression analysis model for long-term prognosis. Measurement data were presented as mean ± standard deviation (χ± SD) from three independent replicate experiments. One-way analysis of variance (ANOVA) was used to compare quantitative data among different groups. All statistical analyses were two sided and conducted using SPSS version 17.0 (SPSS 17.0, Chicago, IL). *P*<.05 was considered statistically significant.

## Results

### Expression of ABCC4 and p53 in Pretreatment Biopsy Specimens

**Figure 1:**
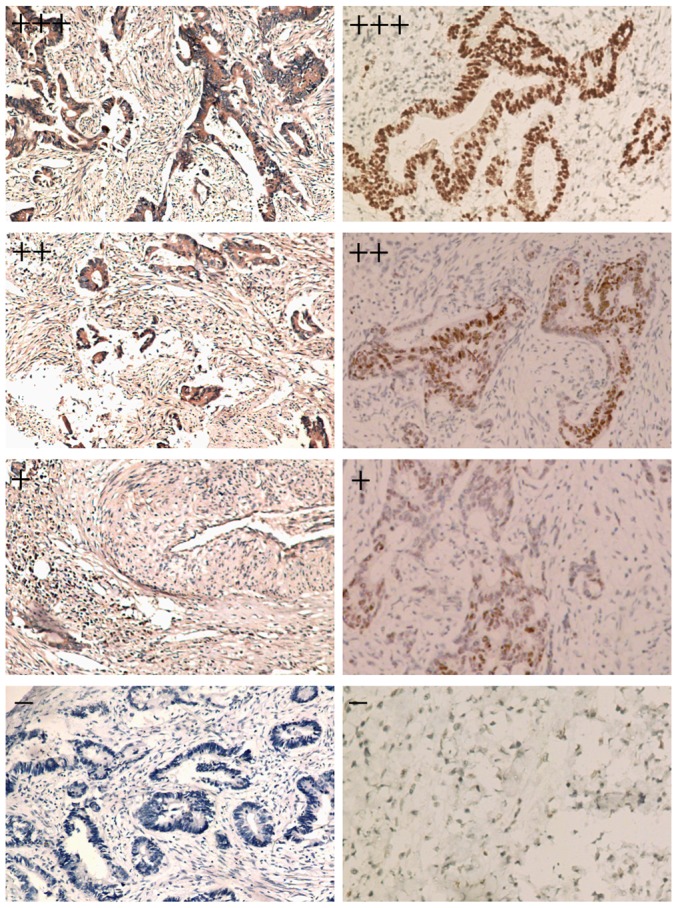
Representative Immunostains to Each Score of ABCC4 and p53 Protein in Pretreatment Biopsy Specimens. **Left**: The expression of ABCC4 protein was mainly cytomembrane and cytoplasm in epithelial rectal cancer cells (×100 magnification); **Right**: The expression of p53 was mainly nuclei in the rectal cancer cells (×100 magnification).

Because pre-treatment biopsy specimens harvested under colonoscopy was not an easy work: not only because the tissue specimens were very small and easily broken to clips, but also for the potential danger of bowel blooding and tumor metastasis due to pincers touch directly to the mass. Therefore, we carefully collected 2–3 biopsy tissue specimens from each case for HE examination to confirm the diagnosis of rectal adenocarcinoma, then used the remaining tissues for immunohistochemical analyses to detect the expression and subcellular localization of ABCC4 and p53. We also obtained 30 pretreatment frozen fresh samples from the 121 patients enough for the analysis of ABCC4 expression by real-time RT-PCR. The representative immunostains of each score are shown in **Figure 1**. The expression of ABCC4 was mainly located in the cytomembrane and cytoplasm, while the expression of p53 was mainly detected in the nuclei of epithelial cancer cells. Meanwhile, 16 cases (13.2%) achieved pathological complete response (pCR) after neoadjuvant radiotherapy, as shown in our previously published paper [5].

### High Expression of ABCC4 and p53 Mutant Predicts a Poor Long-term Prognosis in LARC Patients Receiving Preoperative nRT

A total of 93 patients underwent LAR surgery and the rest 28 cases received APR procedure, the CRM status of every case was considered negative according to postoperative pathology reports, for the shortest distance between the rectal tumor (including noncontiguous tumor) and the mesorectal fascia was ≥1 mm.

We first classified the 121 LARC patients into ABCC4 high-expression group (++/+++; 68/121; 56.2%) and ABCC4 low-expression group (−/+; 53/121; 43.8%) according to immunohistochemistry. ABCC4 mRNA levels in 30 pretreatment frozen fresh samples as measured by real-time RT-PCR assay were consistent with protein expression level measured by immunohistochemistry (r = 0.558, *P* = 0.001). There was no significant difference between ABCC4 expression and any of the clinicopathological factors including age, gender, tumor size, gross type, cell differentiation, serum CEA expression, serum CA19-9 expression, preoperative T stage, N stage and p53 type (χ^2^ test *P*>.05 for all) (**Table 1**).

**Table 1:** Correlation of Clinicopathological Characteristics with ABCC4 Expression in 121 LARC Patients Treated with Preoperative nRT.

Characteristics	Cases (n)	Expression of ABCC4 (n)		*P* value
		+/− (n = 53)	++/+++(n = 68)	
Age				1.00
≤50 y	37	16	21	
>50 y	84	37	47	
Gender				.275
Female	59	29	30	
Male	62	24	38	
Tumor size				.461
≤5 cm	48	19	29	
>5 cm	73	34	39	
Gross type				1.00
Mass	30	13	17	
Ulcerative	91	40	51	
Differentiation				.366
Well	21	10	11	
Moderate	72	34	38	
Poor	28	9	19	
CEA				.275
≤10 ng/ml	66	32	34	
>10 ng/ml	55	21	34	
CA19-9				.464
≤37 U/ml	58	23	35	
>37 U/ml	63	30	33	
T stage				.558
T3	83	38	45	
T4	38	15	23	
N stage				.392
N-	92	38	54	
N+	29	15	14	
p53 type				.856
Wild	58	26	32	
Mutant	63	27	36	

Abbreviations: ABCC4, ATP-binding cassette subfamily C member 4; CA19-9, carbohydrate antigen 19-9; CEA, carcinoembryonic antigen; LARC, locally advanced rectal carcinoma; nRT, neoadjuvant radiotherapy.

**Figure 2:**
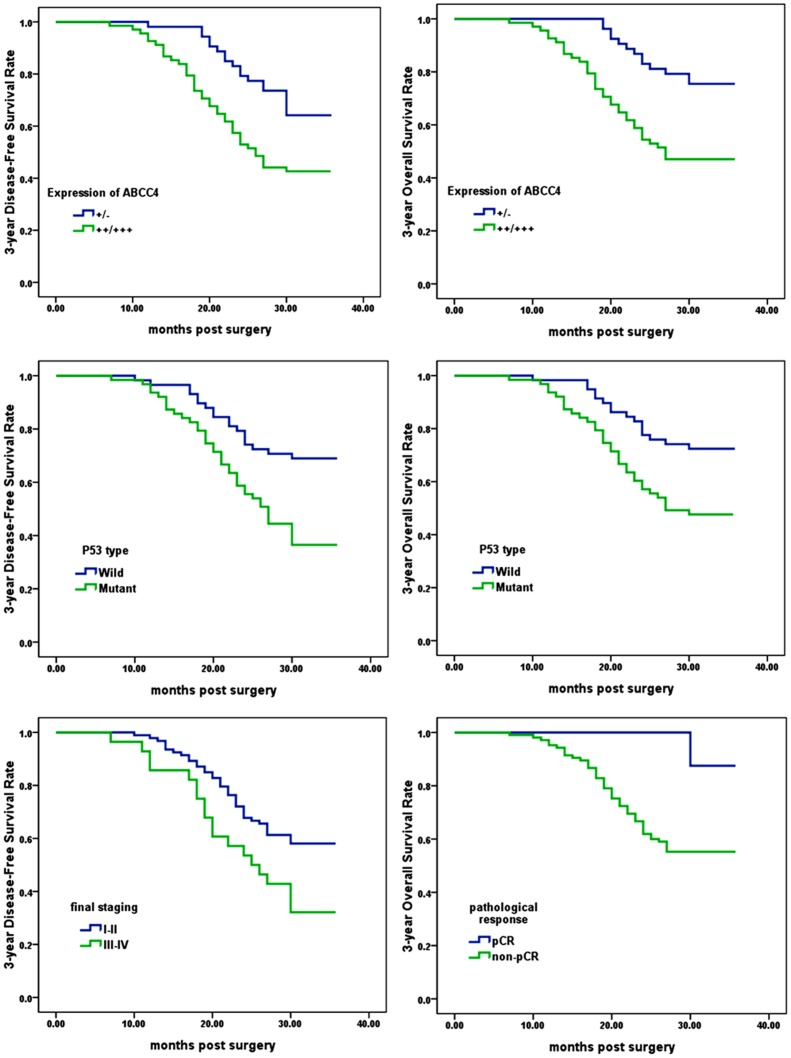
Kaplan–Meier analysis of Factors Associated with Prognosis in LARC Patients Receiving Preoperative nRT. **Right**: 3-year overall survival was significantly shorter in the patients with high ABCC4 expression (*P* = .001), those with p53 mutation (*P* = .004), and those with poor pathological response (*P* = .017) than the corresponding counterparts; **Left**: 3-year disease-free survival rate was significantly shorter in the patients with ABCC4 expression (*P* = .005), those with p53 mutation (*P* = .001), and those with high final tumor staging (*P* = .011) than the corresponding counterparts. *P* values were from log-rank test.

The mean postoperative follow-up duration was 34.5 (SD = 8.6) months. **Figure 2** presents clinicopathological factors significantly related to prognosis in Kaplan-Meier models. Multivariate Cox proportional hazards analysis further indicated that high expression of ABCC4 and p53 mutant in pretreated specimens and poor pathological response were significantly associated with unfavorable 3-year OS (*P* = .036, *P* = .047, and *P* = .044, respectively); while high expression of ABCC4 and p53 mutant in pretreated specimens and high final tumor staging were also significantly associated with worse 3-year DFS (*P* = .027, *P* = .019, and *P* = .032, respectively) (**Table 2**). Age (*P* = .974 for OS; *P* = .873 for DFS), gender (*P* = .807 for OS; *P* = .802 for DFS), tumor size (*P* = .431 for OS; *P* = .471 for DFS), gross type (*P* = .873 for OS; *P* = .576 for DFS), cell differentiation (*P* = .679 for OS; *P* = .475 for DFS), serum CEA expression (*P* = .142 for OS; *P* = .501 for DFS), serum CA19-9 expression (*P* = .558 for OS; *P* = .606 for DFS), preoperative T stage (*P* = .467 for OS; *P* = .722 for DFS), N stage (*P* = .474 for OS; *P* = .145 for DFS) and surgical procedures (*P* = .678 for OS; *P* = .856 for DFS) were not significantly associated with long-term prognosis in those receiving preoperative nRT.

**Table 2:** Multivariate Cox Regression Analysis of Predictive Factors for Long-Term Prognosis in 121 LARC Patients Treated with Preoperative nRT.

Variables	3-year OS		3-year DFS	
	HR (95% CI)	*P*	HR (95% CI)	*P*
Expression of ABCC4 (++/+++ vs. +/−)	1.469(1.025–2.105)	.036	1.513(1.049–2.183)	.027
P53 type (mutant vs. wild)	1.441(1.005–2.066)	.047	1.535(1.072–2.199)	.019
final staging (III–IV vs. I–II)	_	_	1.607(1.042–2.478)	.032
pathological response (non-pCR vs. pCR)	1.452(1.010–2.089)	.044	_	_

Abbreviations: ABCC4, ATP-binding cassette subfamily C member 4; CI, confidence interval; DFS, Disease-Free Survival; HR, hazard ratio; LARC, locally advanced rectal carcinoma; nRT, neoadjuvant radiotherapy; OS, Overall Survival; pCR, pathological complete response.

### Down-regulation of ABCC4 Expression in RKO-KD Cells by RNA Interference

**Figure 3:**
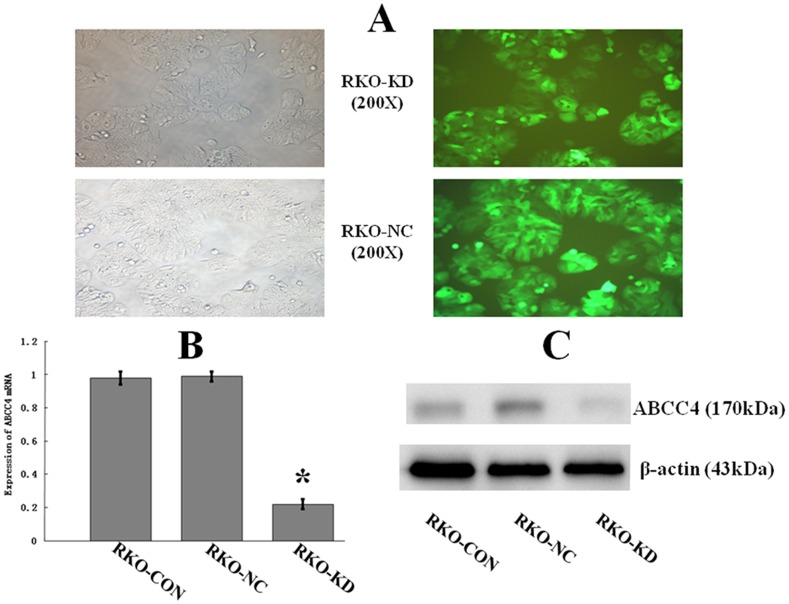
Down-regulation of ABCC4 Expression in RKO-KD Cell Line by RNA Interference. **A**: The shRNA construct containing the green fluorescent protein gene indicated successful transfection via lentivirus. The highest infection efficiency was detected at multiplicities of infection of 5 for RKO-KD (upper) and RKO-NC (lower) cells. (left panel as background control, ×200 magnification). **B**: Real-time RT-PCR showed that ABCC4 expression in RKO-KD was significantly lower than that in RKO-NC and RKO-CON (*P<.05; Error bars, SD). **C**: Western Blot assay showed that the expression of ABCC4 protein was significantly decreased in RKO-KD than in RKO-NC and RKO-CON (*P*<.05). No difference in ABCC4 protein between RKO-NC and RKO-CON.

As shown in **Figure 3**, the highest lentivirus infection efficiency was detected at multiplicities of infection of 5. Ninety-six hrs after the infection, green fluorescent protein was expressed in >90% of both RKO-KD and RKO-NC cells. Real-time RT-PCR and Western blot assay showed that the expression level of ABCC4 mRNA and protein in RKO-KD cells were significantly lower than those in RKO-NC or RKO-CON cells. The expression levels of ABCC4 mRNA and protein in RKO-NC cells were consistent with those in RKO-CON cells. Thus, the shRNA construct worked efficiently in silencing ABCC4 expression in RKO cells.

### Down-regulation of ABCC4 Significantly Enhanced Irradiation-induced Suppression of Tumor Growth in Xenograft Model

**Figure 4:**
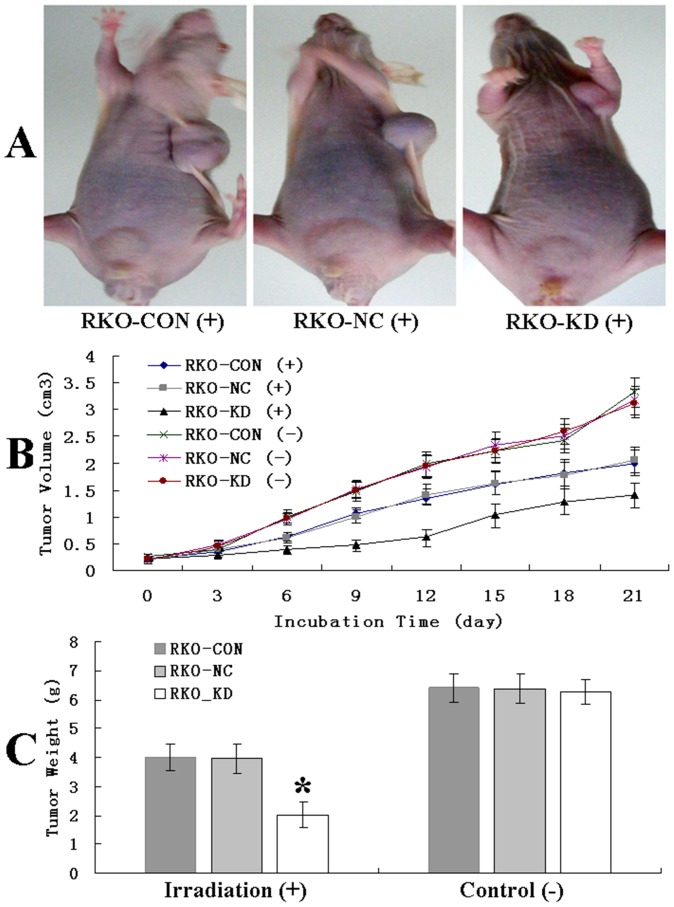
Down-regulation of ABCC4 Expression Significantly Enhanced Irradiation-induced Suppression of Tumor Growth in Xenograft Model. A: Three representative mice following a total dose of 10Gy irradiation. B: Tumor volume curve (cm^3^) in xenograft models of RKO cells with or without ABCC4 knockdown following irradiation (*P*<.05; Error bars, SD) or not (*P*>.05) in a time-dependent manner. C: The mean tumor weight (g) in xenograft models of RKO cells with or without ABCC4 knockdown following irradiation (*P<.05 for both; Error bars, SD) or not (*P*>.05) at the end of the observation.

The tumor growth following the irradiation is presented in **Figure 4**. Compared to the controls, tumor growth in RKO-KD xenograft model was significantly inhibited in a time-dependent manner following the irradiation (*P*<.05). The mean tumor weight (g) of xenograft model, measured at the end of the observation, was significantly lower in RKO-KD model 2.01 (±0.18) % than those in RKO-NC model 3.97 (±0.21) % and RKO-CON model 4.01 (±0.19) % (*P*<.05 for each comparison). There were no significant differences in tumor volume and weight among the three groups without the irradiation (*P*>.05).

### Down-regulation of ABCC4 Expression Enhanced Intracellular cAMP Accumulation and Arrested Cell Cycle Following Irradiation

**Figure 5:**
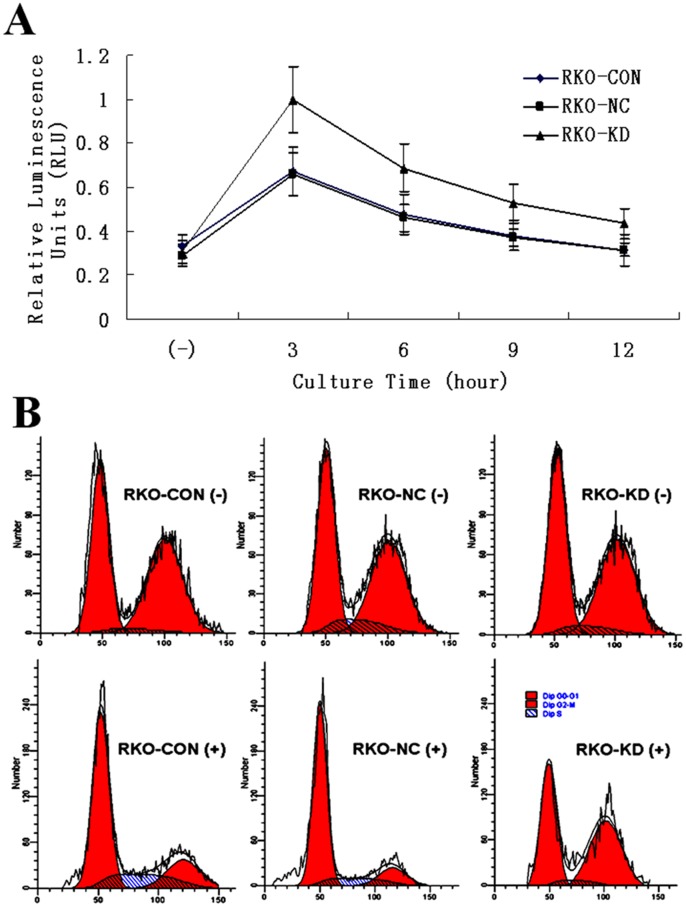
Intracellular cAMP Concentration and Cell Cycle Arrest Following 4 Gy Irradiation. **A**: Curves of intracellular cAMP changes in RKO cells with or without ABCC4 knockdown (*P*<.05; Error bars, SD). **B**: The proportion of cell cycle arrested at the G0/G1 phase in RKO cells with or without ABCC4 knockdown (*P*<.05); while there was no significant difference among the three cell groups without irradiation (*P*>.05).

As shown in **Figure 5A**, intracellular cAMP immediately accumulated following irradiation, and gradually decreased in a time-dependent manner. The average cAMP concentration was significantly higher in RKO-KD 0.66 (±0.25) % than that of RKO-NC 0.45 (±0.15) % and RKO-CON 0.46 (±0.16) % after exposed to 4 Gy irradiation (*P*<.05).

The proportion of cells arrested at the G0/G1 phase accounted for 43.54 (±3.07) % in RKO-KD, which was significantly lower than that in RKO-NC 65.73 (±3.78) % and in RKO-CON 66.52 (±3.64) %, following a single dose of 4 Gy irradiation (*P*<.05 for each comparison). There was no significant difference in cell cycle distribution among the three cell groups without irradiation (*P*>.05 for each comparison) (**Figure 5B**).

## Discussion

In this study, we firstly demonstrated that high expression of ABCC4 and p53 mutant type were significant predictive factors of poor long-term prognosis in LARC patients receiving preoperative nRT. Interestingly, ABCC4 expression and P53 mutant were also positively correlated with short-term response to nRT according to tumor regression grading by postoperative histological examinations in our previous study [5]. The 3-year OS and 3-year DFS were the common measurements to evaluate long-term effect of therapy [14]. It is widely accepted that TNM stage, cell differentiation, regional lymph nodes and serum tumor marker expression are associated with long-term prognosis of rectal carcinoma [15]–[16]. However, to date there have been few biomarkers proven to be effective in determining both short-term response to nRT and long-term prognosis in LARC patients.

The present neoadjuvant therapy model for locally advanced rectal cancer patients combines both 5-FU-based chemotherapy and radiotherapy [17]. Therefore, the greatest advantage of using ABCC4 as a novel biomarker in predicting radioresistance lies on that the most ideal molecular marker should be associated with response to both irradiation and cytotoxic drug. The chemoresistance property of ABCC4 has been reported in several studies on various carcinomas including CRC [18]. To the best of our knowledge, this is the first study investigating both the predictive and prognostic value of ABCC4 in radioresistance. ABCC4 appears as a promising marker in the selection of LARC patients for neoadjuvant therapy, thus improving prognosis.

p53 is known to be a key mediator in regulating cell functions, such as cell proliferation, apoptosis, and DNA repair [19]. p53 type is associated with the response to radiotherapy in several carcinomas [20], whereas the role of p53 type in the prognosis of LARC patients treated with nRT has not been reported. Wild-type p53 protein is very unstable, susceptible to hydrolysis, and its half-life is only a few minutes; while mutated p53 protein is relatively stable, the half-life of which reaches several hours. Therefore, immunohistochemistry method is only able to detect the expression of mutant-type p53 protein, but can’t detect the expression of wild-type p53 protein. In the present study, we firstly proved that p53 mutant type predicted a poor prognosis in a large number of LARC patients treated with nRT for a long-term follow-up. Although ABCC4 expression and p53 mutant type were independently associated with short-term response to nRT and long-term prognosis of LARC patients in our study, previous researches have presented immunohistochemical data showing overexpression of multidrug resistance-associated protein is related to aberrant p53 expression in CRC [21]. It is therefore of great importance for further studies to elucidate the molecular mechanisms of the correlation between ABCC4 expression and p53 mutant.

There was no significant difference among the three mice groups in *in vivo* experiment without irradiation (**Figure 4**). It is consistent with our previous control experiment *in vitro* that there were no significant differences in cell proliferation capacity without irradiation [5]. These results suggested that ABCC4 conferred the responses to irradiation, rather than affect the growth and apoptosis of CRC cells.

The molecular mechanism of radioresistance is complex and remains largely unclarified. Several biological factors including inherent cellular radiosensitivity, the distribution of cell cycle and tumor hypoxia are considered to be important for radio-response. The antioxidant enzyme within the mitochondria and cell cycle-dependent kinase actually play important roles in the detoxification of free radicals generated by cellular metabolism and environmental/therapeutic irradiation [22]–[23].

In this study, we found that down-regulation of ABCC4 expression enhanced intracellular cAMP accumulation following irradiation (**Figure 5A**), indicating that ABCC4 had an important efflux property in the regulation of intracellular concentrations of cAMP. This finding is supported by a previous study indicating that extrusion of intracellular second messengers such as cAMP and cGMP were ATP-dependent and modulated by ABCC4 in various cell types [24]. cAMP is a key effector of G-protein-coupled receptors that regulates multiple aspects of cell processes, especially in cell signaling conduction [25]. In addition, accumulation of cAMP mediates a wide range of cellular responses to numerous extracellular stimuli including irradiation through activating cAMP dependent protein kinase signalling pathways [26], thereby controlling a wide range of cellular activities such as cell apoptosis, proliferation, and differentiation [27]. It is likely that ABCC4 acts as a mediator of cAMP-dependent signal transduction to the nucleus [28].

Cell cycle is one of the determinative factors in response to irradiation [29]. There has been a consensus that an important cellular response to DNA-damaging agents induced by ionizing radiation is the activation of G1-S cell cycle checkpoint [30], which initiates signals that can ultimately activate G1-S checkpoint to arrest cell cycle at G0/G1 phase and provide enough time to repair DNA damage induced by irradiation [31]. Our results of cell cycle distribution following irradiation (**Figure 5B**) indicated that more cells in RKO-NC and RKO-CON group were arrested at the G0/G1 phase before G1-S checkpoint to allow DNA repair; while a large fraction of cells in RKO-KD group lost this important checkpoint that can protect cells from irradiation induced apoptosis, thus accumulating in the G2 phase, which appears to be most radiosensitive during the whole cell cycle [32]. These results suggest that the role of ABCC4 in resistance to irradiation may also be related to cell cycle checkpoints. Even though S phase appeared in every other phase, it only accounted for a small proportion and didn’t cause significant difference among groups in statistical analysis.

It should be emphasized here that molecular mechanism of chemoresistance is largely different from radioresistance. Cytotoxic drugs work in a continued, slow and systematic way, therefore allow sufficient time for the change of key proteins in apoptosis signaling pathway, which could well explain the potential mechanism of chemoresistance; however, as to irradiation, which delivers an instantaneous high energy directly to interior of cancer cells, generates amount of reactive oxygen species inducing DNA damage. The apoptosis signaling pathway may not be the key determinants. Herein, we identified that down-regulation of ABCC4 expression enhanced intracellular cAMP accumulation and noticeable deficiency of G1-S phase checkpoint in cell cycle following irradiation, which might help in explaining the mechanism by which radiation induced apoptosis of CRC cells with decreased ABCC4 expression by shRNA knockdown.

In conclusion, the present study demonstrated that ABCC4 is an important molecule in regulating the response to irradiation both *in vitro* and *in vivo*, and is significantly associated with a poor prognosis in LARC patients who received preoperative nRT. These data support that ABCC4 is a useful predictive marker for radioresistance and a novel therapeutic target for improving prognosis in LARC patients treated with preoperative nRT, which may be important in selecting patients for individualized radiotherapy to improve life quality of LARC patients.

## References

[1] Benson AB 3rd, Bekaii-Saab T, Chan E, Chen YJ, Choti MA, et al (2012) Rectal cancer. J Natl Compr Canc Netw 10: 1528–1564. 23221790 10.6004/jnccn.2012.0158

[2] van Gijn W, Marijnen CA, Nagtegaal ID, Kranenbarg EM, Putter H, et al (2011) Preoperative radiotherapy combined with total mesorectal excision for resectable rectal cancer: 12-year follow-up of the multicentre, randomised controlled TME trial. Lancet Oncol 12: 575–582. 21596621 10.1016/S1470-2045(11)70097-3

[3] Willett CG, Duda DG, di Tomaso E, Boucher Y, Ancukiewicz M, et al (2009) Efficacy, safety, and biomarkers of neoadjuvant bevacizumab, radiation therapy, and fluorouracil in rectal cancer: a multidisciplinary phase II study. J Clin Oncol 27: 3020–3026. 19470921 10.1200/JCO.2008.21.1771 PMC2702234

[4] Fu CG, Tominaga O, Nagawa H, Nita ME, Masaki T, et al (1998) Role of p53 and p21/WAF1 detection in patient selection for preoperative radiotherapy in rectal cancer patients. Dis Colon Rectum 41: 68–74. 9510313 10.1007/BF02236898

[5] Yu ZQ, Zhang C, Wang H, Lao XY, Chai R, et al (2013) Downregulation of ATP-binding cassette subfamily C member 4 increases sensitivity to neoadjuvant radiotherapy for locally advanced rectal carcinoma. Dis Colon Rectum 56: 600–608. 23575399 10.1097/DCR.0b013e31827c2b80

[6] Wittgen HG, van den Heuvel JJ, Krieger E, Schaftenaar G, Russel FG, et al (2012) Phenylalanine 368 of multidrug resistance-associated protein 4 (MRP4/ABCC4) plays a crucial role in substrate-specific transport activity. Biochem Pharmacol 84: 366–373. 22542979 10.1016/j.bcp.2012.04.012

[7] Marquez B, Ameye G, Vallet CM, Tulkens PM, Poirel HA, et al (2011) Characterization of Abcc4 gene amplification in stepwise-selected mouse J774 macrophages resistant to the topoisomerase II inhibitor ciprofloxacin. PLoS One 6: e28368. 22162766 10.1371/journal.pone.0028368 PMC3230599

[8] Huynh T, Norris MD, Haber M, Henderson MJ (2012) ABCC4/MRP4: a MYCN-regulated transporter and potential therapeutic target in neuroblastoma. Front Oncol 2: 178. 23267433 10.3389/fonc.2012.00178 PMC3526013

[9] Russel FG, Koenderink JB, Masereeuw R (2008) Multidrug resistance protein 4 (MRP4/ABCC4): a versatile efflux transporter for drugs and signalling molecules. Trends Pharmacol Sci 29: 200–207. 18353444 10.1016/j.tips.2008.01.006

[10] Lin-Lee YC, Tatebe S, Savaraj N, Ishikawa T, Tien Kuo M (2001) Differential sensitivities of the MRP gene family and gamma-glutamylcysteine synthetase to prooxidants in human colorectal carcinoma cell lines with different p53 status. Biochem Pharmacol 61: 555–563. 11239498 10.1016/s0006-2952(00)00592-x

[11] Li X, Tan X, Yu Y, Chen H, Chang W, et al (2011) D9S168 microsatellite alteration predicts a poor prognosis in patients with clear cell renal cell carcinoma and correlates with the down-regulation of protein tyrosine phosphatase receptor delta. Cancer 117: 4201–4211. 21387281 10.1002/cncr.26028

[12] Chang W, Wu L, Cao F, Liu Y, Ma L, et al (2011) Development of autoantibody signatures as biomarkers for early detection of colorectal carcinoma. Clin Cancer Res 17: 5715–5724. 21771877 10.1158/1078-0432.CCR-11-0199

[13] Ji-Fu E, Xing JJ, Hao LQ, Fu CG (2012) Suppression of lung cancer metastasis-related protein 1 (LCMR1) inhibits the growth of colorectal cancer cells. Mol Biol Rep 39: 3675–3681. 21732059 10.1007/s11033-011-1142-2

[14] Sargent D, Shi Q, Yothers G, Van Cutsem E, Cassidy J, et al (2011) Two or three year disease-free survival (DFS) as a primary end-point in stage III adjuvant colon cancer trials with fluoropyrimidines with or without oxaliplatin or irinotecan: data from 12,676 patients from MOSAIC, X-ACT, PETACC-3, C-06, C-07 and C89803. Eur J Cancer 47: 990–996. 21257306 10.1016/j.ejca.2010.12.015 PMC3073413

[15] Caricato M, Borzomati D, Ausania F, Valeri S, Rosignoli A, et al (2006) Prognostic factors after surgery for locally recurrent rectal cancer: an overview. Eur J Surg Oncol 32: 126–132. 16377120 10.1016/j.ejso.2005.11.001

[16] Jakob C, Aust DE, Liebscher B, Baretton GB, Datta K, et al (2011) Lymphangiogenesis in regional lymph nodes is an independent prognostic marker in rectal cancer patients after neoadjuvant treatment. PLoS One 6: e27402. 22087309 10.1371/journal.pone.0027402 PMC3210168

[17] Engstrom PF, Arnoletti JP, Benson AB 3rd, Chen YJ, Choti MA, et al (2009) NCCN Clinical Practice Guidelines in Oncology: rectal cancer. J Natl Compr Canc Netw 7: 838–881. 19755047 10.6004/jnccn.2009.0057

[18] Gradilone A, Pulcinelli FM, Lotti LV, Trifirò E, Martino S, et al (2008) Celecoxib upregulates multidrug resistance proteins in colon cancer: lack of synergy with standard chemotherapy. Curr Cancer Drug Targets 8: 414–420. 18690847 10.2174/156800908785133178

[19] Muller PA, Vousden KH (2013) p53 mutations in cancer. Nat Cell Biol 15: 2–8. 23263379 10.1038/ncb2641

[20] Lu C, El-Deiry WS (2009) Targeting p53 for enhanced radio- and chemo-sensitivity. Apoptosis 14: 597–606. 19259822 10.1007/s10495-009-0330-1

[21] Fukushima Y, Oshika Y, Tokunaga T, Hatanaka H, Tomisawa M, et al (1999) Multidrug resistance-associated protein (MRP) expression is correlated with expression of aberrant p53 protein in colorectal cancer. Eur J Cancer 35: 935–938. 10533474 10.1016/s0959-8049(99)00035-0

[22] Candas D, Fan M, Nantajit D, Vaughan AT, Murley JS, et al (2013) CyclinB1/Cdk1 phosphorylates mitochondrial antioxidant MnSOD in cell adaptive response to radiation stress. J Mol Cell Biol 5: 166–175. 23243068 10.1093/jmcb/mjs062 PMC3656610

[23] Nantajit D, Fan M, Duru N, Wen Y, Reed JC, et al (2010) Cyclin B1/Cdk1 phosphorylation of mitochondrial p53 induces anti-apoptotic response. PLoS One 5: e12341. 20808790 10.1371/journal.pone.0012341 PMC2925892

[24] Sassi Y, Abi-Gerges A, Fauconnier J, Mougenot N, Reiken S, et al (2012) Regulation of cAMP homeostasis by the efflux protein MRP4 in cardiac myocytes. FASEB J 26: 1009–1017. 22090316 10.1096/fj.11-194027 PMC3289499

[25] Witkowski G, Rola R, Szulczyk P (2012) Effect of cyclic adenosine monophosphate on the G protein-dependent inward rectifier K(+)-like channel current in medial prefrontal cortex pyramidal neurons. J Physiol Pharmacol 63: ; 457–462. 23211299

[26] Kim C, Cheng CY, Saldanha SA, Taylor SS (2007) PKA-I holoenzyme structure reveals a mechanism for cAMP-dependent activation. Cell 130: 1032–1043. 17889648 10.1016/j.cell.2007.07.018

[27] Haste NM, Talabani H, Doo A, Merckx A, Langsley G, et al (2012) Exploring the Plasmodium falciparum cyclic-adenosine monophosphate (cAMP)-dependent protein kinase (PfPKA) as a therapeutic target. Microbes Infect 14: 838–850. 22626931 10.1016/j.micinf.2012.05.004 PMC3967591

[28] Copsel S, Garcia C, Diez F, Vermeulem M, Baldi A, et al (2011) Multidrug resistance protein 4 (MRP4/ABCC4) regulates cAMP cellular levels and controls human leukemia cell proliferation and differentiation. J Biol Chem 286: 6979–6988. 21205825 10.1074/jbc.M110.166868 PMC3044954

[29] Jorgensen TJ (2009) Enhancing radiosensitivity: targeting the DNA repair pathways. Cancer Biol Ther 8: 665–670. 19287209 10.4161/cbt.8.8.8304

[30] Langerak P, Russell P (2011) Regulatory networks integrating cell cycle control with DNA damage checkpoints and double-strand break repair. Philos Trans R Soc Lond B Biol Sci 366: 3562–3571. 22084383 10.1098/rstb.2011.0070 PMC3203453

[31] Zhao L, Bode AM, Cao Y, Dong Z (2012) Regulatory mechanisms and clinical perspectives of miRNA in tumor radiosensitivity. Carcinogenesis 33: 2220–2227. 22798379 10.1093/carcin/bgs235 PMC3483015

[32] Pawlik TM, Keyomarsi K (2004) Role of cell cycle in mediating sensitivity to radiotherapy. Int J Radiat Oncol Biol Phys 59: 928–942. 15234026 10.1016/j.ijrobp.2004.03.005

